# Scabies in Military and Civil Life

**Published:** 1919

**Authors:** 


**Scabies in Military and Civil Life. Its Differentiation, Complications and Treatment.** By F. C. Knowles, Captain M. R. C., U. S. Army. *Journal of the American Medical Association,* November 16, 1918.

The author speaks of the occurrence of scabies in military and civil life and gives two tables showing: (1) the differentiation between scabies and pediculosis corporis; (2) scabies in military life and in civil life. During 15 years of civil hospital practice the cases of scabies observed by the author amounted to about 5 o/o of the total number of dermatologic cases, while in 12 months 1,500 of the 2,000 cases treated at a British hospital were either frankly scabies or secondary to scabies.

*Method of Treating Scabies.* Scabies should be treated by the routine suggested by the leading dermatologists of France, Great Britain and the Unites States. The greatest care is exercised in treating scabies in a separate hut or tent, in the thorough disinfection of all articles of clothing, and in the use of separate baths.

*Complications of Scabies.* Complications secondary to scabies are much more frequent and markedly more severe in military than in civil practice. If boils continue to recur or a large number are present, autogenous vaccines are indicated. Septic ulcer and inflammation of the connective tissue not infrequently require rest in bed and the local application of ammoniated mercury.

A rather prolonged convalescence is observed in approximately 10 o/o of scabies in military practice.

